# Mammary gland, kidney and rumen urea and uric acid transporters of dairy cows differing in milk urea concentration

**DOI:** 10.1038/s41598-023-44416-9

**Published:** 2023-10-11

**Authors:** Marie C. Prahl, Carolin B. M. Müller, Klaus Wimmers, Björn Kuhla

**Affiliations:** 1 Research Institute for Farm Animal Biology (FBN), Institute of Nutritional Physiology ‘Oskar Kellner’, Wilhelm-Stahl-Allee 2, 18196 Dummerstorf, Germany; 2 Research Institute for Farm Animal Biology (FBN), Institute of Genome Biology, Wilhelm-Stahl-Allee 2, 18196 Dummerstorf, Germany

**Keywords:** Molecular biology, Physiology

## Abstract

The milk urea concentration (MUC) serves as indicator of urinary nitrogen emissions, but at comparable crude protein (CP) intake, cows with high (HMU) and low (LMU) MUC excrete equal urea amounts. We hypothesized that urea and uric acid transporters and sizes of the kidney, mammary gland, and rumen account for these phenotypes. Eighteen HMU and 18 LMU Holstein dairy cows fed a low (LP) and normal (NP) CP diet were studied. Milk, plasma and urinary urea concentrations were greater with NP feeding, while plasma and urinary urea concentrations were comparable between phenotypes. Milk and plasma uric acid concentrations were higher with LP feeding but not affected by phenotype. The milk-urine uric acid ratio was greater in HMU cows. The mRNA expressions of the ruminal urea transporter *SLC14A1* and *AQP10*, the mammary gland and rumen *AQP3*, and the mammary gland uric acid transporter *ABCG2* were not affected by group or diet. Renal *AQP10,* but not *AQP3*, *AQP7*, and *SLC14A2* expressions, and the kidney weights were lower in HMU cows. These data indicate that renal size and *AQP10* limit the urea transfer from blood to urine, and that MUC determines if uric acid is more released with milk or urine.

## Introduction

Livestock farming faces growing pressure, as it is responsible for 60% of global ammonia (NH_3_) emissions and 23% of global nitrous oxide (N_2_O) emissions^1^. While NH_3_ endangers human and animal health due to its toxicity and causes damage to forests and buildings, N_2_O is a greenhouse gas possessing a 267-fold higher warming potential than CO_2_ over the next 100 years. The major factor determining NH_3_ emissions is the excretion of urinary urea, which is hydrolyzed to NH_3_ and carbon dioxide by microbial urease excreted with feces^2^. According to their size and numbers, cattle, in particular dairy cows excrete the largest amounts of urine and feces as compared with other farm animals. The dietary crude protein (CP) intake is directly correlated with urinary urea excretions^3,4^. Therefore, an adequate CP supply, respectively, is a pivotal in feeding practices of dairy cows influencing environmental pollutions.

The majority of dietary CP is degraded in the rumen by microbes forming NH_3_, which in turn is predominantly used for the synthesis of microbial protein. Another portion of the ruminal NH_3_ pool, however, is transported from the ruminal lumen through the rumen epithelium into the portal vein blood^5^. The absorbed NH_3_ is detoxified by the liver resulting in the formation of urea, which in turn is subsequently transported in the circulation to various organs. It is known that blood urea enters the mammary gland compartments to be secreted with milk, hence the blood urea concentration is positively correlated with milk urea concentration (MUC)^6,7^. As a role, as higher the CP intake, as higher the MUC. Therefore, MUC is often used as an indicator of proper CP intake^8^. Besides, urea is transported with the blood to the kidney from which it is excreted with urine. It has been proposed that MUC reflects the urinary urea excretion^2,8^. However, dairy cows with intrinsically high MUC (HMU) had higher plasma urea concentrations but comparable urinary urea excretion as compared to dairy cows with low MUC (LMU) despite comparable milk yield and feeding the same diet^3,9^. Moreover, we have previously shown that HMU compared to LMU cows have a worse urea as well as uric acid renal clearance rate^3^, the latter facilitating higher plasma urea and uric acid concentrations, respectively. The lower renal clearance in HMU cows could be related to osmolytic factors, e.g. polar substances such as uric acid^10^. Furthermore, specific mechanisms play a role in the excretion of uric acid from the blood, such as the transport by the solute carrier family 22 member 12 (*SLC22A12*), or the synthesis or uric acid in the kidney by xanthine dehydrogenase (*XDH*). In addition, HMU cows might simply have a smaller kidney organ size which limits the excretion rate. The latter assumption is supported by the observation that the nitrogen content of the diet influences the weight of various organs, including the weight of kidneys^11^. A further reason for the divergent MUC phenotypes could be due to differences in urea metabolism. While LMU and HMU cows do not differ in hepatic urea metabolism^12^, LMU cows have greater abundances of ureolytic bacteria in their rumen^13^. Hence, differences in urea recycling mechanisms involving the transport between the blood stream and the mammary gland, between blood and kidney, or across the rumen epithelium may further account for the divergent phenotype of LMU and HMU cows. As a high-polar molecule, urea possesses a low permeability through lipid bilayers and as such it is transported carrier-mediated through the epithelium^5^. The urea transport is facilitated by specific transport proteins expressed in various organs, including the kidney^14,15^, the mammary gland, and the rumen epithelium^11,16,17^. More specifically, the urea transporter A (UT-A), encoded by the *SLC14A2* gene, occurs in six alternative splice forms, all evidenced in the kidney of non-ruminant species and involved in the concentration of urine^18,19^. However, the role of *SLC14A2* in renal urea excretion in cattle is far from clear^20,21^. A further urea transporter (UT-B) is encoded by the *SLC14A1* gene and predominantly expressed in the rumen where it facilitates the rapid transport across rumen papillae into the lumen^20^. Besides solute carriers, a subgroup of the aquaporin water channel family, namely the aquaglyceroporins are permeable to water, glycerol, and urea. This subgroup involves *AQP3*, *AQP7* and *AQP10*, which are all expressed in the rumen wall and responsive to changing dietary CP concentrations^16,17^. Whether different expression of urea transporters and aquaporins account for high and low milk urea secretions in dairy cows while feeding the same ration is not known. Therefore, the aim of this study was to investigate the mRNA expression of genes encoding the urea transporters in the mammary gland, the kidney and the rumen wall as well as renal and mammary gland uric acid transporters and organ weights of dairy cows with intrinsic high and low MUC.

## Materials and methods

### Animals and experimental design

The animal experiment was evaluated by the ethical body of and approved by the State Department for Agriculture, Food Security and Fisheries Mecklenburg-Western Pomerania, Rostock, Germany (LALLF permission no. 7221.3-1-052/17) and was conducted in accordance with the relevant regulations of the authority and the ARRIVE guidelines (https://www.arriveguidelines.org). Thirty-six non-pregnant German Holstein cows from second to fourth late lactation were selected from two commercial farms based on their milk yield and MUC. The animals were obtained in pairs of one with high (HMU: 276 ± 4 mg/L; *n* = 18) and one with low (LMU: 186 ± 4 mg/L; *n* = 18) MUC, but with a comparable milk yield of 32.5 ± 0.9 kg/d. Cows were transported to the free-ranging barn of the experimental facilities at FBN (Dummerstorf, Germany) in 9 blocks, each consisting of two LMU and HMU cows. The cow pairs within one block entered the trial at different times, resulting in 18 sub-blocks. LMU and HMU groups had comparable lactation numbers (each 2.7). Cow pairs of each sub-block underwent a two-week adaptation period at the free-ranging barn. Cow pairs received alternately a total mixed ration with normal (NP: 15.9 ± 0.1%) or low (LP: 13.8 ± 0.2%) CP but comparable metabolizable energy (ME) content of 10.1 ± 0.1 MJ/kg of dry matter (DM) (Table 1). Isoenergetic rations were formulated by increasing the starch concentration of the LP relative to the NP ration. Animals of the four groups (HMU-NP, HMU-LP, LMU-NP and LMU-LP; *n* = 9 cows in each group) were fed at 0500 h and 1700 h, had ad libitum access to feed and water and were milked at 0430 h and 1630 h. After the two-week adaptation period, animals had a comparable milk yield of 23.4 ± 0.8 kg/d and were 329 ± 13 days in milk. Cows were transferred to tie-stalls in a climate-controlled room (constant 15 °C) and continuously fed the same diet. On day 4 before morning feeding and again on day 8 two h after the morning feeding, a rumen fluid (750 mL) sample was obtained using an esophageal probe connected to a vacuum pump. Samples were instantaneously analyzed for pH and NH_3_ concentrations. On day 8, cows were implanted a jugular vein catheter and equipped with a urinal, which was connected with a flexible plastic tube (4.5 diameter) to a 30 L-container^22^. On day 9, urine was collected without acidification, a sample was taken and stored at -20 °C for later analyses. On day 10 at 10:00 h and 19:00 h, and again on day 11 at 07:00 h, a blood sample was taken from the jugular catheter in a 9-mL EDTA-containing tube (S-Monovetten; Sarstedt, Nürnbrecht, Germany), centrifuged at 1345 × *g* for 20 min at 4 °C, and the obtained plasma was stored at − 80 °C.

**Table 1:** Feed constitutes, nutrient composition and energy concentration of the normal protein (NP) and low protein (LP) diets (means ± SEM).

Parameter	NP	LP
Ingredients g/kg of DM		
Grass silage	275 ± 14	227 ± 13
Corn silage	311 ± 4	369 ± 11
Triticale silage	22.2 ± 15.2	–
Forage rye silage	14.2 ± 9.7	82.5 ± 25.4
Hay	17.5 ± 7.7	–
Barley straw	4.0 ± 2.7	4.3 ± 3.0
Corn meal	52.9 ± 5.6	70.8 ± 10.5
Wheat seeds	116 ± 12	131 ± 6
Rapeseed extraction meal	143 ± 11	100 ± 7
MF 2000^1^	28.5 ± 19.4	–
Mineral feed^2^	9.1 ± 0.3	10.3 ± 0.3
Limestone^3^	3.8 ± 0.1	3.7 ± 0.2
Feed salt^4^	1.5 ± 0.1	1.6 ± 0.0
Nutrients, g/kg of DM		
Crude ash^5^	74 ± 4	69 ± 1
Crude fat	29 ± 1	27 ± 1
Crude protein	159 ± 1	139 ± 2
ADF	197 ± 4	189 ± 3
NDF	377 ± 7	353 ± 5
Starch	210 ± 6	248 ± 5
Sugar	23.0 ± 1.4	14.4 ± 1.6
DM content, %	39 ± 1	38 ± 1
ME, MJ/kg DM	10.2 ± 0.1	10.1 ± 0.2
NEL, MJ/kg DM	6.2 ± 0.1	6.2 ± 0.1
Utilizable crude protein^6^	152 ± 1	146 ± 1
N, g/kg DM^7^	29.5 ± 0.6	25.1 ± 0.3

^1^MF2000 pell. (Ceravis Produktion und Transport GmbH, Malchin, Germany): composition: 24% crude protein, 2.6% crude fat, 5.1% crude fiber, 8% crude ash, 0.73% calcium, 0.5% phosphorus, 0.65% sodium, 7.1 MJ NEL/kg; Additives: 10,000 I.E. vitamin A, 1125 I.E. vitamin D3, 40 mg vitamin E, 0.6 mg I, 0.4 mg Co, 50 mg Mn, 75 mg Zn, 0.4 mg Se.

^2^Panto Mineral R 8609 (HL Hamburger Leistungsfutter GmbH, Hamburg, Germany): composition: 20% calcium, 6% phosphorous, 8% sodium, 6% magnesium, 0.03% inorganic nitrogen, 13.74% phosphorous pentoxide. Additives per kg original substance: 900,000 IU vitamin A, 200,000 IU vitamin D3, 4.5 g vitamin E, 1.5 g Cu, 8 g Zn, 5 g Mn, 60 mg I, 21 mg Co, 50 mg Se.

^3^Bergophor CaCO3 V001 (Hohburg Mineralfutter GmbH, Lossatal, Germany): 37% calcium.

^4^Animal feed salt (ESCO—European Salt Company GmbH & Co.KG, Hanover, Germany): 38% sodium, 0.3% calcium, 0.01% magnesium.

^5^Measured quantity elements g/kg in LP: calcium 7.0 ± 0.2, phosphorous 4.1 ± 0.1, sodium 2.3 ± 0.2, magnesium 2.3 ± 0.1, potassium 10.3 ± 0.6; NP: calcium 7.5 ± 0.4, phosphorous 4.4 ± 0.1, sodium 2.4 ± 0.2, magnesium 2.6 ± 0.1, potassium 10.5 ± 0.6

^6^Utilizable crude protein (g/kg DM) = [11.93 – (6.82 × UDP) (g/kg DM)/crude protein (g/kg DM)] × ME (MJ/kg DM) + 1.03 × UDP (g/kg DM), with UDP = undegradable protein (GfE, 2001).

^7^N measured in fresh feed including volatile nitrogen compounds and normalized to dry matter content.

From day 10–12, the container for urine collection was prefilled with 400 mL (564 g) of 50% sulfuric acid and were kept on a shaker or magnetic stirrer. The excreted urine volume was determined daily and acidified urine samples were taken and stored at − 20 °C. Milking was performed at 0630 h and 1830 h and subsamples from the evening and morning milking were pooled according to the respective milk yield. Fresh pooled milk samples were sent off for major constitute analysis and another aliquot stored at − 20 °C.

On day 13 after morning feeding and milking, animals were transferred to the institute’s slaughterhouse. The body weight was measured and animals were stunned by a captive bolt stunning. During the subsequent exsanguination, a blood sample was collected to obtain EDTA-plasma as described above. The obtained plasma was stored at -80 °C until analysis. The kidneys, the mammary gland and the emptied and rinsed reticulorumen were weighed and tissue samples were taken. Samples from the left renal cortex, the left hind mammary gland quarter and papillae from the ventral rumen were placed on ice, cut into small pieces, snap frozen in liquid N_2_ and stored at − 80 °C until further analysis.

### Ammonia analysis in rumen fluid samples

Rumen fluid samples were analyzed for NH_3_ concentrations according to the Conway method^23^. Briefly, a Conway flask was filled with 5 mL reagent solution (5 g boric acid dissolved in 200 mL ethanol and 300 mL distilled water) and 10 mL Conway-indicator solution (33 mg bromocresol green and 66 mg methyl red in 100 mL ethanol). Then, 1 mL rumen fluid and 1 mL saturated potassium carbonate solution were filled into a diffusion insert before closing the flask. After 24 h of incubation at room temperature, the solution was titrated with 1 N hydrochloric acid until the color changed from green to pink.

### Analyses in feed samples

Dry matter (DM) content of feed samples were determined by air drying for 24 h at 60 °C and for 4 h at 105 °C, followed by grinding and chemical analysis of nutrient composition by the accredited laboratory of Landwirtschaftliche Untersuchungs- und Forschungsanstalt der LMS Agrarberatung GmbH ( LUFA GmbH, Rostock, Germany) (Table 1). The metabolizable energy (ME) content was calculated based on the recommendations by the German Society of Nutrition Physiology^24^. The ME intake (MEI) was calculated as follows: MEI (MJ of ME/d) = ME (MJ/kg of DM) × DMI. Frozen fresh feed samples were ground with the application of dry ice and were analyzed for N by LUFA GmbH using the Kjedahl method.

### Analyses in milk, urine and plasma samples

Fresh milk samples were sent to the State Inspection Association for Performance and Quality Testing Mecklenburg-Western Pomerania e.V. (LKV Güstrow, Germany) for analysis of milk protein, fat, and lactose by mid-infrared spectroscopy (MilkoScan; Foss GmbH, Rellingen, Germany). Frozen milk samples were thawed and centrifuged for 10 min at 4 °C and 50.000 × *g* to detach the fat from skim milk as described previously^3^. Plasma samples collected on day 10 at 10:00 h and 19:00 h, on day 11 at 07:00 h, and on the day of slaughter were thawed, pooled in equal shares. The plasma pool and skim milk were analyzed for urea and uric acid concentrations using ABX Pentra C400 analyzer (HORIBA Europe GmbH, Oberursel, Germany) and the kit LT-UR0010 (urea; Labor + Technik Eberhard Lehmann GmbH, Berlin, Germany) and A11A01-670 (uric acid, HORIBA ABX SAS, Montpellier, France). The measured skim milk concentrations were recalculated for whole milk. Acidified urine samples were 50-fold diluted and analyzed for urea by HPLC (1200/1260 infinity II Series; Agilent) with a 300 × 7.8 mm Rezex RCM-Monosaccharide column (Phenomenex Inc.) as described earlier^3^. The tenfold diluted non-acidified urine was analyzed for uric acid concentration by HPLC as described by Müller et al.^3^ but with the following modifications: separation was performed on a 250 × 4.6 mm Synergi 4 µm Hydro-RP 80 Å column protected by a corresponding 4 × 3 mm pre-column (both Phenomenex Inc., Aschaffenburg, Germany) and the analyte was detected at 230 nm on a UV detector.

Renal clearance rates for urea (RUCR) and uric acid (RUACR) were calculated as as previously described by Spek et al.^25^

RUCR (L/min) = Urea_Urine_ (mg/d)/Urea_Plasma_ (mg/L)/1440 (min/d);

RUACR (L/min) = UricAcid_Urine_ (mg/d)/UricAcid_Plasma_ (mg/L)/1440 (min/d), Similarly, the urea transfer rate into milk (UTM) and the uric acid transfer rate into milk (UATM) were calculated as follows:

UTM (L/d) = Urea_Milk_ (mg/d)/Urea_Plasma_ (mg/L);

UATM (L/d) = UricAcid_Milk_ (mg/d)/UricAcid_Plasma_ (mg/L).

### RNA extraction and RT-qPCR

RNA was extracted from 18 to 20 mg tissue powder using innuPREP RNA Mini Kit 2.0 and remaining DNA was digested with innuPREP DNase I Digest Kit (both Analytik Jena AG, Jena, Germany). RNA concentrations were measured spectrophotometrically using a NanoPhotometer (Implen GmbH, Munich, Germany). Quality of the RNA was determined based on the RNA integrity number (RIN) factors, which were measured on an Agilent 2100 Bioanalyzer (Agilent Technologies Inc., Santa Clara, CA), yielding RIN factors for kidney > 7.9, for mammary gland > 7.4 and for rumen papillae > 7.9. For cDNA synthesis, 1000 ng total RNA was reverse transcripted with Sensifast cDNA Synthesis Kit (Bioline, London, UK) using a Thermocycler (pegstar 96 × HPL, VWR International, Pennsylvania, USA). Real-time qPCR was performed on a LightCycler 2.0 (Roche, Basel, Switzerland) with SensiFAST SYBR No-ROX Kit (Bioline) using 2 µL of cDNA and the primers listed in Supplemental Table 1. If not published in the following references^17,26–29^, primer sequences were deduced using the online Primer3web tool (version 4.1.0). Each cDNA sample was analyzed in duplicate. The efficiency of amplification was calculated with LinRegPCR software version 2014.4 (Academic Medical Centre, Amsterdam, Netherlands). Amplicons were analyzed on an ABI 3130 Genetic Analyzer (Life Technologies GmbH, Darmstadt, Germany) to confirm sequence identity. Amplicon abundances were quantified using qbasePlus software (Biogazelle, Gent, Belgium) normalized to the reference genes eukaryotic translation initiation factor-3 subunit K (*EIF3K*^30^*)* and peptidylprolyl isomerase A (*PPIA*^31^) for rumen villi, and PPIA and emerin (*EMD*^32^) for kidney and mammary gland.

### Statistical analysis

The required sample size was calculated iteratively using CADEMO^33^, which based on a two-factorial variance analysis including MUC and CP as fixed factors. The minimum sample size for each group was *n* = 9 setting a type-I error α = 0.05, type-II error β = 2.0, residual variance σ^2^ = 1, and effect size d = 1. Statistical analyses were performed using the SAS software (version 9.4, SAS Institute Inc., Cary, NC, USA). Data were analyzed using the MIXED procedure with a confidence interval of 0.95, an unstructured covariance structure (TYPE = UN option), and a degrees of freedom approximation according to Kenward-Roger. Data from two animals fed the NP diet were excluded from statistical analysis, due to a change in feed intake caused by technical problems with climate control. Therefore, 8 HMU-NP, 9 HMU-LP, 8 LMU-NP, and 9 LMU-LP cows were included in the statistical analysis. The sub-blocks 1–14 formed block 1–7, and the remaining three sub-blocks were summarized in block 8. Sub-blocks could not be considered in the model because of over-parameterization. The ANOVA model included the fixed factors MUC (HMU/LMU), diet (NP/LP), the interaction of MUC × diet, and as a random factor the block of sampling (1–8). The assumptions of the MIXED procedure were checked for each variable and the normality of dependent variables was tested according to Shapiro–Wilk, included in the UNIVARIATE procedure of SAS. Normality was violated for some dependent variables. However, linear mixed-effects models are remarkably robust to violations of normality^34^. Thus, we refrained from transforming variables to achieve normality. The statistical model was designed as follows:

$$ {\text{y}}_{{{\text{ijkl}}}} = \mu + {\text{a}}_{{\text{i}}} + \beta_{{\text{j}}} + \gamma_{{\text{k}}} + \left( {\beta \gamma } \right)_{{{\text{jk}}}} + {\text{e}}_{{{\text{ijkl}}}} $$

y_ijkl_: response variable, µ: average test score, a_i_: independent N(0; σ^2^_a_)-distributed random effect of block on level i, β_j_: fixed effect of diet on level j, γ_k_: fixed effect of MUC on level k, (βγ)_jk_: two-times interaction between diet on level j and MUC on level k, e_ijkl_: independent N(0; σ^2^_ijkl_)-distributed experimental error term

For each fixed effect the least-square means (LSM) and their standard error (SE) were calculated. To perform a partition analysis of the LSM for the interaction of MUC × diet, the slice statement of the MIXED procedure was used. Furthermore, the Tukey–Kramer procedure was used to assay the pairwise differences. Pearson correlation coefficients were calculated using the CORR procedure in SAS. Significance was defined at a *P*-value < 0.05 and tendencies were defined at 0.05 < *P* < 0.1. Results are presented as LSM ± SEM unless stated otherwise.

## Results

### Animal characteristics

Cows with divergent MUC did not differ in dry matter intake independent of the diet (Table 2). Cow groups had comparable CP intake, but animals on the NP diet ingested 436–566 g more CP per day than on the LP diet (*P* < 0.001). Milk yield, as well as milk lactose and protein concentrations were not affected by diet or MUC. However, milk fat concentration was on average 4.5 g per kg milk higher in HMU than LMU cows (*P* < 0.05).

**Table 2:** Animal characteristics, intake and milk composition of dairy cows with high (HMU) and low (LMU) milk urea concentration (MUC) fed a diet containing a normal (NP) and a low crude protein level (LP) under conditions of interval feeding (97% of ad libitum intake).

Parameter	NP				LP				*P*-value^1^		
	HMU	LMU	SE	*P*-value^2^	HMU	LMU	SE	*P*-value^2^	Diet	MUC	Diet × MUC
BW, kg	650	690	32	0.246	722	736	35	0.671	0.075	0.257	0.574
DMI, kg/d	17.6	17.0	0.9	0.429	16.4	16.5	0.8	0.900	0.228	0.622	0.507
MEI, MJ/d	178	171	8	0.465	168	170	8	0.858	0.471	0.681	0.513
CP intake, g/d	3180^ac^	3060^ab^	141	0.444	2614^bd^	2630^ cd^	140	0.913	< 0.001	0.629	0.527
Water intake, L/d	62^a^	60^ab^	7	0.548	51^b^	58^ab^	7	0.062	0.069	0.376	0.086
Milk yield, kg/d	24.9	23.4	9.2	0.457	21.6	22.2	9.2	0.709	0.222	0.773	0.426
Milk fat, g/kg	50^a^	43^b^	4	0.010	47^ab^	45^ab^	4	0.353	0.947	0.013	0.181
Milk protein, g/kg	40	37	2	0.162	38	38	2	0.927	0.592	0.334	0.277
Milk lactose, g/kg	47	46	1	0.511	47	48	1	0.676	0.347	0.847	0.446

Data are given as least square means and standard error (SE).

^a,b,c,d^Different superscript letters within one row indicate *P* < 0.05 (Tukey-test).

^1^*P*-value from ANOVA analysis.

^2^*P*-value from Tukey slice test.

### Urea and uric acid concentrations and transfer rates

According to the experimental design, MUC was 37–67 mg /L higher in HMU cows (*P* < 0.01), and were on average 88 mg/L higher on the NP than LP diet (*P* < 0.001; Table 3). Milk uric acid concentrations tended to be higher in HMU than LMU cows and increased with decreasing dietary CP content (*P* < 0.05). Urinary urea concentrations were on average 45% higher on the NP compared to the LP diet (*P* < 0.01), and this effect was particularly apparent in LMU cows, who had 62% higher urinary urea concentration on the NP than LP diet (*P* < 0.01). In contrast, urinary uric acid concentration was on average 23% lower on the NP than LP diet (*P* < 0.05), and this difference was particularly explained by a 30% reduction in urinary uric acid concentration when HMU cows received the NP compared to the LP diet (*P* < 0.05). However, urinary urea and urinary uric acid concentrations were not affected by MUC. Plasma urea concentration tended to be on average 11% higher in HMU than LMU cows (*P* < 0.1) and was 23% higher on the NP than LP diet (*P* < 0.01). The plasma uric acid concentration tended to be 11% higher on the LP than NP diet (*P* < 0.1).

**Table 3:** Milk, urine and plasma urea concentrations, ruminal pH and ammonia concentrations of dairy cows with high (HMU) and low (LMU) milk urea concentration (MUC) fed a diet containing a normal (NP) and a low crude protein level (LP) under conditions of interval feeding (97% of ad libitum intake). Data are given as least square means and standard error (SE).

Parameter	NP				LP				*P*-value^1^		
	HMU	LMU	SE	*P*-value^2^	HMU	LMU	SE	*P*-value^2^	Diet	MUC	Diet × MUC
Milk urea, mg/L	365^a^	298^b^	43	0.010	262^bc^	225^ac^	44	0.115	< 0.001	0.004	0.362
Milk uric acid, mg/L	14.8^ab^	12.0^a^	1.9	0.139	17.6^ab^	16.0^b^	1.8	0.392	0.038	0.100	0.612
Urinary urea, g/L	15.9^ab^	16.7^a^	2.2	0.633	12.2^ab^	10.3^b^	2.3	0.243	0.004	0.643	0.251
Urinary uric acid, g/L	0.32^a^	0.38^ab^	0.07	0.312	0.46^b^	0.46^ab^	0.07	0.978	0.036	0.449	0.471
Urine volume, L/d	15.5	15.4	1.9	0.962	14.1	15.3	1.8	0.402	0.598	0.587	0.541
Plasma urea, mg/L^3^	308^ac^	277^ab^	19	0.137	250^bd^	227^ cd^	18	0.219	0.004	0.058	0.802
Plasma uric acid, mg/L^3^	5.6	5.8	0.6	0.700	6.3	6.4	0.6	0.977	0.092	0.764	0.794
Urinary urea/ milk urea, mg/mg	45	55	8	0.073	47	47	7	0.935	0.609	0.203	0.168
Urinary uric acid/ milk uric acid, mg/mg	23^a^	40^b^	11	0.003	25^ab^	27^a^	11	0.628	0.278	0.012	0.051
RUCR^3^, L/min	0.59	0.66	0.11	0.386	0.52	0.54	0.11	0.784	0.198	0.413	0.655
RUACR^3^, L/min	0.54	0.69	0.14	0.211	0.64	0.74	0.14	0.388	0.451	0.137	0.743
UTM^3^, L/d	30^a^	26^ab^	6	0.062	24^b^	24^ab^	6	0.918	0.077	0.148	0.189
UATM^3^, L/d	60	43	8	0.053	56	52	9	0.611	0.717	0.079	0.270
Rumen pH	7.2	7.3	0.1	0.269	7.2	7.2	0.1	0.959	0.814	0.399	0.439
Rumen fluid NH_3_, mmol/L	7.9	6.2	0.9	0.085	6.2	5.6	1.0	0.525	0.163	0.093	0.398

^a,b,c,d^ different superscript letters within one row indicate *P* < 0.05 (Tukey-test).

^1^*P*-value from ANOVA analysis.

^2^*P*-value from Tukey slice test.

^3^NP-HMU: *n* = 8; NP-LMU: *n* = 8; LP-HMU: *n* = 9; LP-LMU: *n* = 8. RUCR, renal urea clearance rate; RUACR renal uric acid clearance rate; UTM, urea transfer rate into milk; UATM, uric acid transfer rate into milk.

However, there were no group effects for plasma uric acid concentrations. Irrespectively, we found significant correlations between plasma and milk urea and plasma and urinary urea concentrations (Table 4). Furthermore, the amount of urea and uric acid secreted with milk or excreted with urine showed decent correlation coefficients with the respective plasma concentration. In addition, strong correlations existed between milk und urinary urea concentrations, whereas the correlation coefficients between the amount of urea or uric acid, respectively, secreted with milk and excreted with urine were weaker.

**Table 4:** Pearson correlation coefficients between milk, urine and plasma urea and uric acid concentrations as well as between the amounts of urea and uric acid secreted with milk or excreted with urine of dairy cows, irrespective of grouping and crude protein feeding.

	Plasma–milk	Plasma–urine	Milk–urine
Urea concentration	0.79	0.59	0.71
	< 0.001	< 0.001	< 0.001
Uric acid concentration	0.55	–	–
	< 0.001	n.s	n.s
Urea amount	0.62	0.52	0.38
	< 0.001	< 0.001	< 0.05
Uric acid amount	0.51	–	0.38
	< 0.005	n.s	< 0.05

Corresponding *P*-values are shown below coefficients.

The pH and NH_3_ concentration in rumen fluid did not differ between diets (Table 3). However, HMU cows tended to have higher ruminal NH_3_ concentrations than LMU cows, particularly on the NP diet (*P* < 0.1). The renal urea (RUCR) and uric acid (RUACR) clearance rates did not differ between HMU and LMU cows or diets. The urea transfer into milk (UTM) tended to be 17% higher in NP than in LP fed cows (*P* < 0.1), while the uric acid transfer rate into milk (UATM) remained unaffected by crude protein intake. On the NP diet, HMU cows tended to have 15% higher UTM and 40% higher UATM than LMU cows (*P* < 0.1). The latter difference is reflected by a tending smaller urine urea: milk urea ratio (*P* < 0.1) and a smaller urine uric acid : milk uric acid ratio in HMU cows, particularly when fed the NP ration (*P* < 0.01).

### Organ weights and mRNA expression

**Figure 1:**
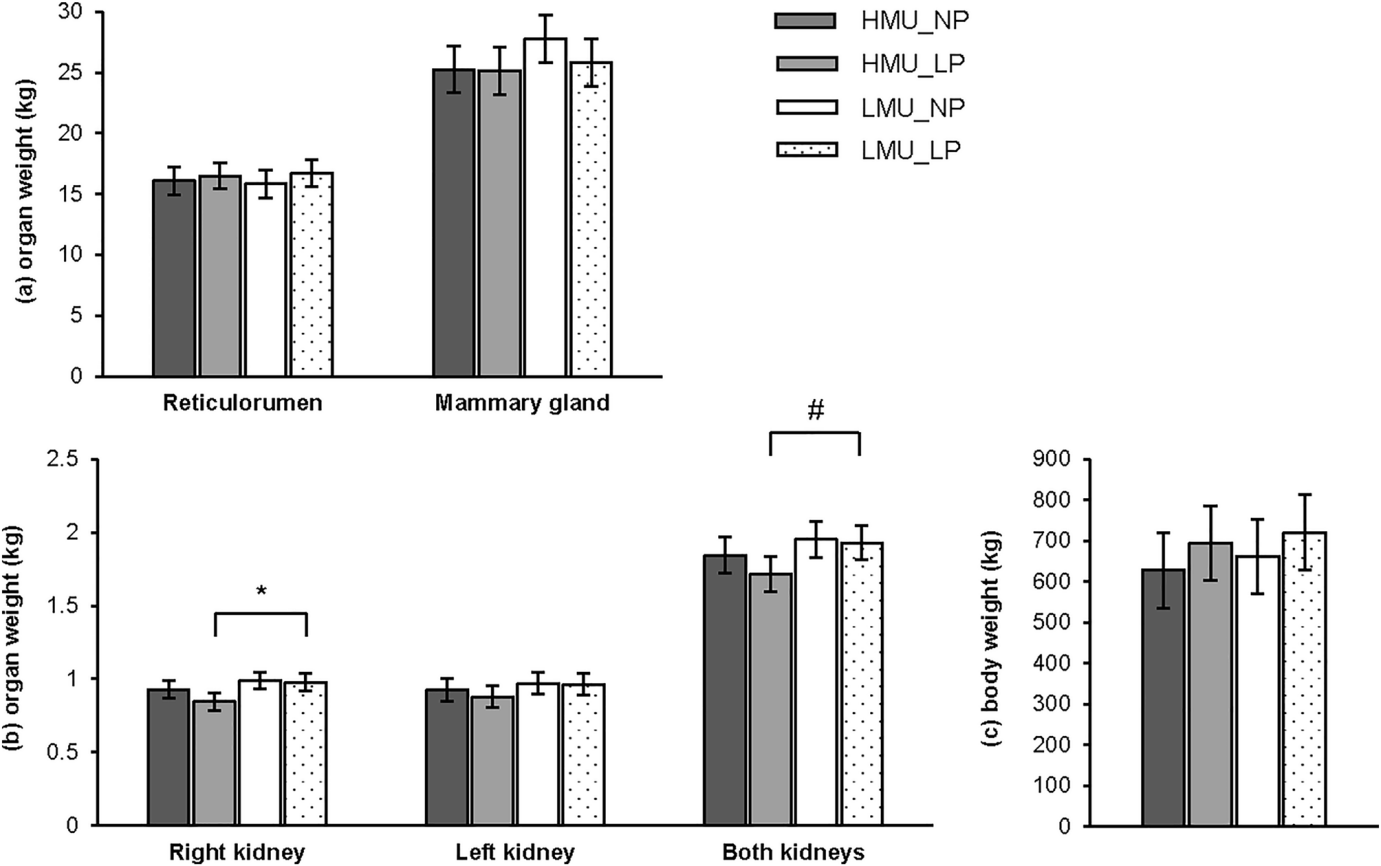
Weights of the reticulorumen and the mammary gland (**a**), Weights of the right, left and both kidneys (**b**), and body weight at the day of slaughter of cows with divergent milk urea concentration, fed a diet with normal (NP) or low (LP) crude protein content. *Indicates *P* < 0.05, # indicates *P* < 0.1; Tukey–Kramer.

The body weight at the day of slaughter was not affected by group or diet. The weight of the right kidney was 9% (*P* < 0.05) and the total kidney weight tended to be 10% lower (*P* < 0.1) in HMU compared to LMU cows (Fig. 1). The weights of the left kidney, the mammary gland and the reticulorumen did not differ between groups or diets.

**Figure 2:**
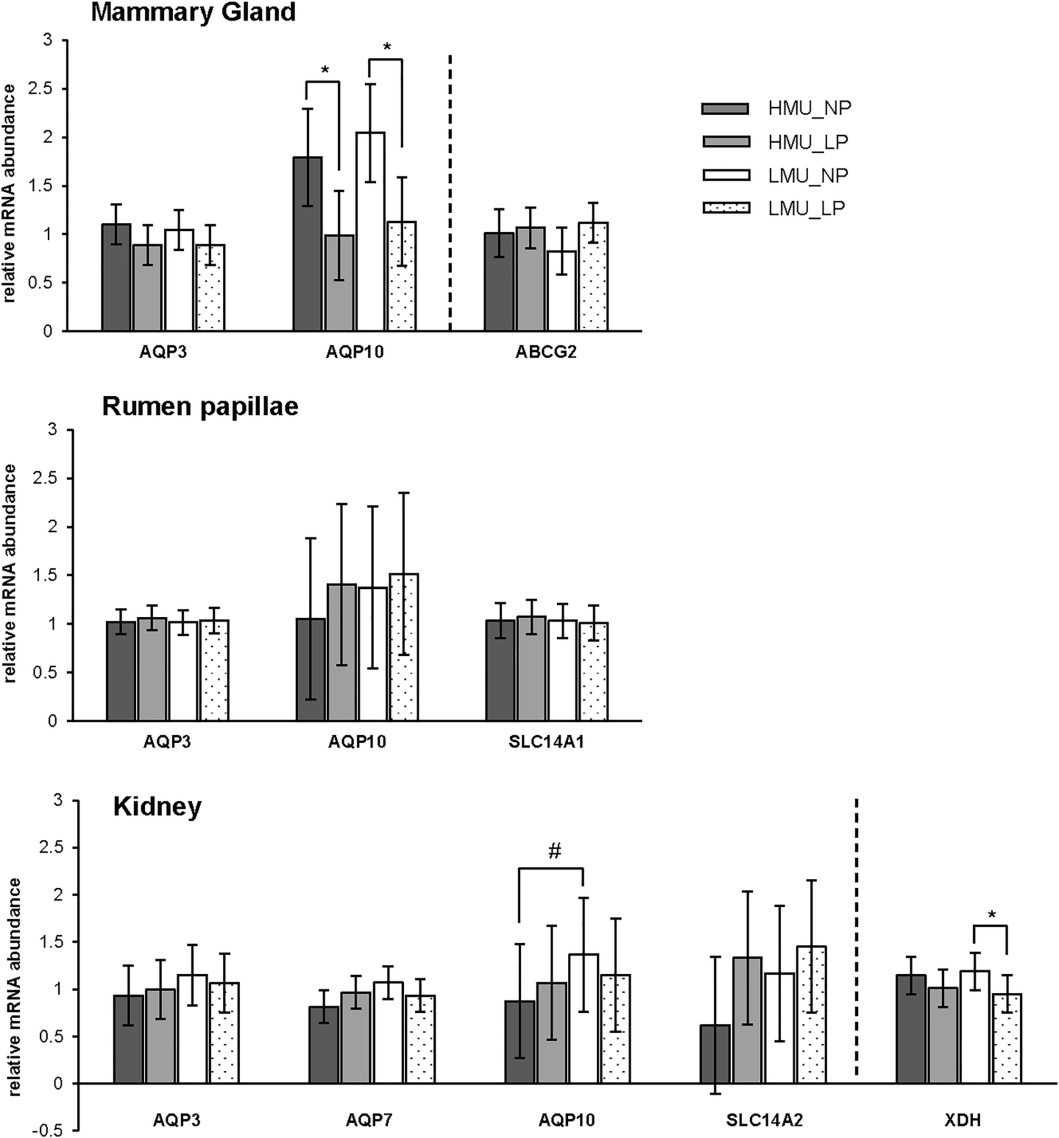
Relative mRNA abundance of the urea transporters aquaporin 3 (*AQP3*), aquaporin 7 (*AQP7*), aquaporin 10 (*AQP10*), urea transporter A (*SLC14A2*), urea transporter B (*SLC14A1*), as well as the uric acid transporter ATP-binding cassette transporter G2 (*ABCG2*) and the uric acid forming enzyme xanthine dehydrogenase (*XDH*). Expressions were analyzed in dairy cows with high (HMU) and low (LMU) milk urea concentration fed a diet with normal (NP) or low (LP) crude protein content. *Indicates *P* < 0.05; Tukey–Kramer.

The mRNA expression of the urea transporter *AQP10* in the kidney tended to be 57% higher in LMU than HMU animals, but only when cows were fed the NP diet (*P* < 0.1; Fig. 2). The relative transcript abundance of *SLC14A2*, *AQP3* and *AQP7* was not different between groups or diets. However, the expression of *AQP10* in the mammary gland rose with increasing dietary CP content (*P* < 0.01; Table 3). Group or diet did not affect the transcript abundance of mammary gland *AQP3*. Similarly, the mRNA expression of *AQP3*, *AQP10* and *SLC14A1* in the rumen papillae did not differ between groups and diets.

To assess, if the divergent urinary-milk uric acid ratio between LMU and HMU cows could be affected by differences in renal uric acid formation, we analyzed the mRNA abundances of the renal uric acid transporter *SLC22A12* and the uric acid forming enzyme *XDH.* While the renal mRNA abundance of *SLC22A12* was below the detection limit, we found renal *XDH* mRNA 25% higher expressed on the NP than on the LP diet in LMU cows. However, there were no group differences in renal *XDH* mRNA expression. Likewise, the mRNA expression of the mammary gland uric acid transporter ATP-binding cassette transporter G2 (*ABCG2)* did not differ between groups and diets.

## Discussion

### Transfer into milk

According to the experimental design, HMU cows had comparable parity, body weight, feed and water intake, milk yield, and mammary gland weight, but higher MUC than LMU cows. The higher MUC of HMU cows was paralleled by higher plasma urea concentrations, the latter could be due to a greater urea synthesis rate by the liver. In an earlier study, we found comparable mRNA and protein abundances of hepatic enzymes controlling urea production^12^, suggesting that the hepatic urea synthesis rate is not a significant factor underlying higher MUC. Given the strong direct correlation between plasma and MUC in the present study and described earlier^2^, it seems that urea transporters regulating the urea flux do not control the transfer from blood to milk. Although UTM tended to be higher in HMU cows, on the NP diet, the abundances of the mammary gland urea transporters *AQP3* and *AQP10* were comparable between HMU and LMU cows. On the other hand, the transcriptional abundances of *AQP3* and further aquaporins are highly regulated at least during the transition from gestation to lactation of rats and pigs^35,36^. Although the role of individual aquaporins in the mammary gland are far from understood, *AQP3* and *AQP10* seem to be predominantly involved in the regulation of water flux than in the transport of small solutes^37^. Besides, there is also reverse transfer of urea from milk to blood, namely from cistern milk to alveoli milk in dairy cows^6,7^. However, which of the aquaporins or urea transporters regulate the reverse urea transport and if the mRNA expression level corresponds to the functional protein abundance needs to be determined in future studies.

Relative to the LMU group, HMU cows tended to have a higher UATM and milk uric acid concentrations, while the plasma uric acid concentration was comparable between groups. The latter results agree with the finding of an earlier study^3^. However, the correlation coefficients between plasma and milk uric acid concentrations were much weaker than they were for urea concentrations. Accordingly, we conclude that the uric acid transfer from the circulation into milk is differently controlled than the urea transfer. From an in vitro experiment using MDCK-II cells, it has been proposed that *ABCG2* facilitates the transfer of uric acid into milk^38^. In dairy cows, the mammary gland *ABCG2* transporter facilitates the excretion of xenobiotics, drugs, riboflavin, and uric acid into milk^39,40^. However, the mRNA expression of *ABCG2* was not different between groups, although the UATM tended to be higher in HMU than LMU cows on the NP ration. This result suggests that *ABCG2* mRNA expression is rarely involved in regulating the transfer of uric acid from blood into milk. It has been reported that the Y581S polymorphism of the *ABCG2* gene ensures a two-fold higher uric acid transfer from plasma into milk compared to the Y/Y variant^39^. We can only speculate if this polymorphism accounts for the lower urine uric acid: milk uric acid ratio and the trend for the higher UATM in HMU than LMU cows under conditions of NP feeding, however, the analysis of the cow’s *ABCG2* genotype was beyond the scope of the present study. Besides, it is interestingly to note that the urinary uric acid: milk uric acid ratio was lower and UATM tended to be higher in HMU than LMU cows, particularly on the NP diet. Based on these results we conclude that the level of milk urea secretion affects the way of uric acid excretion or secretion, respectively.

The concentration of milk uric acid increased with decreasing dietary CP content, independent of the grouping, and this finding corresponds to an earlier study^3^. It has been shown that feed energy restriction reduces milk uric acid concentrations^41–43^, but because the energy content of the NP and LP diet as well as the DMI of cows on both diets were comparable, we can exclude energetic reasons underlying the differences in milk urea concentration. In addition, a contribution of *ABCG2* to the higher milk uric acid concentration with LP feeding can also be excluded. Thus, it seems that with decreasing CP content resulting in declining MUC, the concentration of uric acid in milk increases.

### Transfer into urine

Rojen et al.^17^ have shown by infusion experiments in Holstein dairy cows that the higher the arterial urea concentrations the higher the urea clearance rate via the kidneys. However, while HMU cows reveal higher plasma urea concentration, they do not have higher urinary urea concentrations than LMU cows, indicating no general linear relationship between plasma and urinary urea concentrations. In fact, the correlation coefficient between plasma and urinary urea concentrations over all animals and diets was only 0.59. We have previously shown that the RUCR of HMU cows was on average 16% lower than in LMU cows, and concluded that HMU cows have worse renal performance presumably due to a different abundance of renal urea transporters^3^. Although RUCR was not significantly different between HMU and LMU groups investigated in the present study, we found a lower urinary urea : milk urea ratio and a lower expression of *AQP10* in the kidney of HMU compared to LMU cows, at least when fed the NP diet. This result suggests that *AQP10* limits the urea transfer from blood to urine of HMU cows and accounts for the disproportional relationship between plasma and urinary urea concentrations in these animals. Moreover, the weight of the right kidney of HMU cows was approximately 9% lower than in LMU cows, at least when cows were fed the LP diet. Thus, less kidney parenchyma could limit urea excretion via the kidney and thus be a further reason for the higher plasma urea concentrations of HMU cows.

Plasma, milk, and urinary urea concentrations are clearly reduced when cows are transferred from the NP to the LP diet, and this effect is independent of the HMU or LMU group.

Isozaki et al.^44^ observed an increased urea reabsorption in the inner medullary collecting ducts of the rat kidney after reducing the dietary protein content from 18–8%. The urea reabsorption process by the inner medullary collecting duct is facilitated by numerous UT-A1 proteins, which can be formed from different *SLC14A2* splice forms^45^. However, we found no differences in the abundance of the overall *SLC14A2* transcripts in the renal cortex of NP and LP fed cows. One reason for the absence of different *SLC14A2* mRNA expression could be the difference in dietary protein content, which with 2% is relatively low compared to the difference in protein levels fed to rats (18 vs 8%^44^). Another reason could be that urea reabsorption is rather controlled on the posttranslational level. Terris et al.^46^ reported that the decrease in dietary protein level from 41 to 15% or 4% is accompanied with the increase in the expression level of the 117-kD but not 97-kD UT-A1 protein in the medullary collecting duct of rats^46^. The two (97 and 117 kDa) monomeric UT-A1 forms occur in different states of glycosylation^47^, suggesting that the increase in urea reabsorption from the inner medullary collecting ducts in response to declining dietary protein levels is regulated by posttranscriptional glycosylation. However, it has been reported that the UT-A1 protein expression in the kidney medulla of lambs was not affected by feeding diets containing 1.6, 2.9 or 4.0% nitrogen^11^, which corresponds to the absence of *SLC14A2* mRNA expression differences observed in the present study. While the 117-kD and 97-kD UT-A1 forms are expressed in the inner medulla, a 55-kD UT-A2 form occurs in the inner stripe of outer medulla^45^. This UT-A2 form is sensitive to vasopressin^45^, but it is not known if it is also regulated with changing dietary protein intake. If so, the relative abundances of the different *SLC14A2* splice forms may change without being detected by the chosen PCR method, which bases on the detection of the sum of various splice forms. A further reason for the absence of *SLC14A2* mRNA expression differences may be the sampling site, which in the present study included the renal cortex but excluding the medulla.

The renal uric acid excretion rate was found not affected by the dietary protein content, although plasma uric acid concentrations tended to be and urinary uric acid concentrations were higher on the LP compared to the NP diet. Contrary to our findings Giesecke et al.^48^ reported that the RUACR varies between 12.7 and 35.2 mmol/d, whereas the plasma uric acid concentration remains relatively stable (34.0 ± 7.4 µmol/L) when dairy cows are fed rations with CP levels ranging between 13.8 and 15% of DM. However, a relationship between dietary CP and plasma or urinary uric acid concentrations was not reported in this study^48^. However, renal uric acid excretion seems not to be influenced by plasma urea concentrations, because Rojen et al.^17^ reported no changes in urinary uric acid excretion after ruminal urea infusion of dairy cows. The uric acid transport across the apical membrane of proximal tubule epithelial kidney cells is facilitated by URAT1, at least in humans and rodents^49^. However, we could not detect *SLC22A12* mRNA expression in the present study, and to the best of our knowledge, no study has reported *SLC22A12* mRNA or protein expressions in ruminants so far. Apart from that, we found renal *XDH* mRNA higher abundant in cows fed the NP than LP diet. While RUACR did not differ between diets, plasma and urinary uric acid concentrations tended to be or were greater with LP than NP feeding. These results suggest that renal uric acid synthesis is activated at reduced plasma uric acid concentrations and maintains RUACR.

### Urea transfer into the rumen

Urea is formed as a product of amino acid degradation and ammonia detoxification in the liver. It is transported via the blood stream into the rumen through the salivary glands and across the rumen wall, where it is cleaved by bacterial ureases into carbon dioxide and NH_3_. The urea transfer rate into the rumen is, among others, controlled by ruminal pH and NH_3_ concentrations^50^. An earlier^3^ and the present study shows that HMU tended to have higher ruminal NH_3_ concentrations, suggesting a greater urea transfer rate from blood into the rumen of HMU cows. The urea transport across the rumen epithelia is facilitated at least by UT-B^21^, whose mRNA expression was described to directly correlate with the increase in blood urea concentration of Holstein calves^51^. Despite divergent plasma urea concentrations, we found no differences in ruminal *SLC14A1* mRNA expression between phenotypes, however, changes in the mRNA abundance alone may not necessarily reflect any changes in UT-B protein expression^52^. On the other hand, the UT-B protein abundance in rumen epithelial cells did not differ in lambs fed rations with a nitrogen content ranging between 1.5 and 4.0%^11^. Unfortunately, we could not measure ruminal UT-B protein abundances in the present study and thus focused on the mRNA analysis of further urea transporters, namely aquaporins. In experiments with calves, it was shown that a significant portion of the urea flux occurs via facilitated diffusion through various aquaporins, particularly AQP3^53,54^. Our results show no significant differences in the mRNA expression of *AQP3* and *AQP10* between groups, indicating that the mRNA abundance of these aquaporins is not responsive to changes in plasma urea concentrations. On the other hand, it has been reported that *AQP3* mRNA is down-regulated in diets containing urea, while the dietary CP concentration did not affect the expression of this channel^16^. Furthermore, Simmons et al.^20^ showed that the *SLC14A1* mRNA and UT-B protein abundances were neither controlled by the CP nor energy concentration of the diet but greater in steers fed an isonitrogenous and isoenergetic silage-based compared to concentrate-based diet. In addition, ruminal UT-B and *AQP3* expressions are upregulated when calves are transferred from milk replacer to solid feed feeding^53^, as well as in cows receiving a diet formulated for the pre-partum compared to post-partum requirements^55^. Overall, it seems that the regulation of the ruminal urea transporters is mainly due to dietary fermentable carbohydrates affecting ruminal conditions, such as pH, CO_2_, and short-chain fatty acid concentration^56^, but not controlled by plasma urea or dietary CP concentrations.

## Conclusion

The results of the present study demonstrate that the renal *AQP10* mRNA abundance as well as the weight of the kidneys limits the urea transfer from blood to urine, thereby increasing the plasma urea concentration of cows with intrinsically high milk urea concentration. Although the selection of cows with low milk urea concentrations would not result in less urinary urea excretion, it would ensure a greater kidney size while reducing ruminal ammonia concentrations. No mammary gland urea transporter could be identified to explain divergent milk urea concentration, indicating that higher milk urea concentrations are predominantly driven by higher plasma urea concentrations. The urea transport across the kidney and the mammary gland epithelium seemed to be influenced by uric acid concentrations, but the level of milk urea secretion affects the way of uric acid excretion or secretion, respectively.

### Supplementary Information

Supplementary Table S1.

## Data Availability

All data generated and analysed are available on request from the corresponding author.

## References

[1] Uwizeye A (2020). Nitrogen emissions along global livestock supply chains. Nat. Food.

[2] Burgos SA, Fadel JG, DePeters EJ (2007). Prediction of ammonia emission from dairy cattle manure based on milk urea nitrogen: Relation of milk urea nitrogen to urine urea nitrogen excretion. J. Dairy Sci..

[3] Müller CBM (2021). Differences between Holstein dairy cows in renal clearance rate of urea affect milk urea concentration and the relationship between milk urea and urinary nitrogen excretion. Sci. Total Environ..

[4] Mutsvangwa T, Davies KL, McKinnon JJ, Christensen DA (2016). Effects of dietary crude protein and rumen-degradable protein concentrations on urea recycling, nitrogen balance, omasal nutrient flow, and milk production in dairy cows. J. Dairy Sci..

[5] Abdoun K, Stumpff F, Martens H (2006). Ammonia and urea transport across the rumen epithelium: A review. Anim. Health Res. Rev..

[6] Spek JW, Dijkstra J, Bannink A (2016). Influence of milk urea concentration on fractional urea disappearance rate from milk to blood plasma in dairy cows. J. Dairy Sci..

[7] Spek JW, Dijkstra J, van den Borne JJGC, Bannink A (2012). Short communication: Assessing urea transport from milk to blood in dairy cows. J. Dairy Sci..

[8] Nousiainen J, Shingfield KJ, Huhtanen P (2004). Evaluation of milk urea nitrogen as a diagnostic of protein feeding. J. Dairy Sci..

[9] Correa-Luna M, Donaghy D, Kemp P, Schutz M, Lopez-Villalobos N (2021). Nitrogen use efficiency and excretion in grazing cows with high and low milk urea nitrogen breeding values. Sustainability-Basel.

[10] Yamakita J (2000). Effect of urine storage on urinary uric acid concentrations. Ann. Clin. Biochem..

[11] Marini JC, Klein JD, Sands JM, Van Amburgh ME (2004). Effect of nitrogen intake on nitrogen recycling and urea transporter abundance in lambs. J. Anim. Sci..

[12] Prahl MC (2022). Hepatic urea, creatinine and uric acid metabolism in dairy cows with divergent milk urea concentrations. Sci. Rep..

[13] Honerlagen H (2022). Ruminal background of predisposed milk urea (MU) concentration in Holsteins. Front. Microbiol..

[14] Klein JD, Blount MA, Sands JM (2011). Urea Transport in the Kidney. Compr. Physiol..

[15] Klein JD, Sands JM (2016). Urea transport and clinical potential of urearetics. Curr. Opin. Nephrol. Hy..

[16] Saccà E (2018). Effect of dietary nitrogen level and source on mRNA expression of urea transporters in the rumen epithelium of fattening bulls. Arch. Anim. Nutr..

[17] Rojen BA, Poulsen SB, Theil PK, Fenton RA, Kristensen NB (2011). Short communication: Effects of dietary nitrogen concentration on messenger RNA expression and protein abundance of urea transporter-B and aquaporins in ruminal papillae from lactating Holstein cows. J. Dairy Sci..

[18] Sands JM, Blount MA (2014). Genes and proteins of urea transporters. Subcell Biochem..

[19] Fenton RA, Chou CL, Stewart GS, Smith CP, Knepper MA (2004). Urinary concentrating defect in mice with selective deletion of phloretin-sensitive urea transporters in the renal collecting duct. P. Natl. Acad. Sci. U.S.A..

[20] Simmons NL (2009). Dietary regulation of ruminal bovine UT-B urea transporter expression and localization. J. Anim Sci..

[21] Stewart GS (2005). UT-B is expressed in bovine rumen: potential role in ruminal urea transport. Am. J. Physiol.-Reg..

[22] Kauffman AJ, St-Pierre NR (2001). The relationship of milk urea nitrogen to urine nitrogen excretion in Holstein and Jersey cows. J. Dairy Sci..

[23] Kenten, R. H. *Modern Methods of Plant Analysis*. Vol. 1, 441 (Springer-Verlag Berlin Heidelberg GmbH, 1956).

[24] GfE (2009). New Equations for Predicting Metabolisable Energy of Compound Feeds for Cattle. Proc. Soc. Nutr. Physiol..

[25] Spek JW, Bannink A, Gort G, Hendriks WH, Dijkstra J (2013). Interaction between dietary content of protein and sodium chloride on milk urea concentration, urinary urea excretion, renal recycling of urea, and urea transfer to the gastrointestinal tract in dairy cows. J. Dairy Sci..

[26] Coyle J, McDaid S, Walpole C, Stewart GS (2016). UT-B urea transporter localization in the bovine gastrointestinal tract. J. Membrane Biol..

[27] Kuzmany A (2011). Expression of mRNA, before and after freezing, in bovine blastocysts cultured under different conditions. Theriogenology.

[28] Sauerwein H (2013). Aquaporin-7 mRNA in adipose depots of primiparous and pluriparous dairy cows: Long-term physiological and conjugated linoleic acid-induced changes. J. Dairy Sci..

[29] Bühler S (2018). Effects of energy supply and nicotinic acid supplementation on serum anti-oxidative capacity and on expression of oxidative stress-related genes in blood leucocytes of periparturient primi- and pluriparous dairy cows. J. Anim. Physiol. an..

[30] Kadegowda AKG (2009). Identification of internal control genes for quantitative polymerase chain reaction in mammary tissue of lactating cows receiving lipid supplements. J. Dairy Sci..

[31] Bonnet M, Bernard L, Bes S, Leroux C (2013). Selection of reference genes for quantitative real-time PCR normalisation in adipose tissue, muscle, liver and mammary gland from ruminants. Animal.

[32] Saremi B, Sauerwein H, Danicke S, Mielenz M (2012). Technical note: Identification of reference genes for gene expression studies in different bovine tissues focusing on different fat depots. J. Dairy Sci..

[33] Rasch, D. in *CADEMO-Handbuch: CADEMO-Version 2.111* Vol. 45 3–47 (2003).

[34] Schielzeth H (2020). Robustness of linear mixed-effects models to violations of distributional assumptions. Methods Ecol. Evol..

[35] Nazemi S (2014). Reciprocity in the Developmental regulation of aquaporins 1, 3 and 5 during pregnancy and lactation in the rat. Plos One..

[36] VanKlompenberg MK, Manjarin R, Donovan CE, Trott JF, Hovey RC (2016). Regulation and localization of vascular endothelial growth factor within the mammary glands during the transition from late gestation to lactation. Domest. Anim. Endocrin..

[37] Mobasheri A, Barrett-Jolley R (2014). Aquaporin water channels in the mammary gland: from physiology to pathophysiology and neoplasia. J. Mammary Gland. Biol. Neoplasia.

[38] Schwabedissen HEM, Kroemer HK (2011). In vitro and in vivo evidence for the importance of breast cancer resistance protein transporters (BCRP/MXR/ABCP/ABCG2). Handb. Exp. Pharmacol..

[39] Otero JA (2015). Effect of bovine ABCG2 polymorphism Y581S SNP on secretion into milk of enterolactone, riboflavin and uric acid. Animal.

[40] Min L (2021). An overview of aflatoxin B1 biotransformation and aflatoxin M1 secretion in lactating dairy cows. Anim. Nutr..

[41] Bjerre-Harpoth V (2012). Metabolic and production profiles of dairy cows in response to decreased nutrient density to increase physiological imbalance at different stages of lactation. J. Dairy Sci..

[42] Pires JAA, Larsen T, Leroux C (2022). Milk metabolites and fatty acids as noninvasive biomarkers of metabolic status and energy balance in early-lactation cows. J. Dairy Sci..

[43] Billa PA, Faulconnier Y, Larsen T, Leroux C, Pires JAA (2020). Milk metabolites as noninvasive indicators of nutritional status of mid-lactation Holstein and Montbeliarde cows. J. Dairy Sci..

[44] Isozaki T, Lea JP, Tumlin JA, Sands JM (1994). Sodium-Dependent net urea transport in rat initial inner medullary collecting ducts. J. Clin. Invest..

[45] Wade JB (2000). UT-A2: a 55-kDa urea transporter in thin descending limb whose abundance is regulated by vasopressin. Am. J. Physiol.-Renal..

[46] Terris J, Ecelbarger CA, Sands JM, Knepper MA (1998). Long-term regulation of renal urea transporter protein expression in rat. J. Am. Soc. Nephrol..

[47] Bradford AD (2001). 97-and 117-kDa forms of collecting duct urea transporter UT-A1 are due to different states of glycosylation. Am. J. Physiol.-Renal..

[48] Giesecke D, Ehrentreich L, Stangassinger M, Ahrens F (1994). Mammary and renal excretion of purine metabolites in relation to energy-intake and milk-yield in dairy-cows. J. Dairy Sci..

[49] Xu LQ, Shi YF, Zhuang SG, Liu N (2017). Recent advances on uric acid transporters. Oncotarget.

[50] Lu ZY (2014). Modulation of sheep ruminal urea transport by ammonia and pH. Am. J. Physiol. Regul. Integr. Comp. Physiol..

[51] Naeem A, Drackley JK, Stamey J, Loor JJ (2012). Role of metabolic and cellular proliferation genes in ruminal development in response to enhanced plane of nutrition in neonatal Holstein calves. J. Dairy Sci..

[52] Ludden PA (2009). Effect of protein supplementation on expression and distribution of urea transporter-B in lambs fed low-quality forage. J. Anim Sci..

[53] Berends H, van den Borne JJGC, Rojen BA, van Baal J, Gerrits WJJ (2014). Urea recycling contributes to nitrogen retention in calves fed milk replacer and low-protein solid feed. J. Nutr..

[54] Walpole ME (2015). Serosal-to-mucosal urea flux across the isolated ruminal epithelium is mediated via urea transporter-B and aquaporins when Holstein calves are abruptly changed to a moderately fermentable diet. J. Dairy Sci..

[55] Rojen BA, Theil PK, Kristensen NB (2011). Effects of nitrogen supply on inter-organ fluxes of urea-N and renal urea-N kinetics in lactating Holstein cows. J. Dairy Sci..

[56] Zhong CL, Long RJ, Stewart GS (2022). The role of rumen epithelial urea transport proteins in urea nitrogen salvage: A review. Anim. Nutr..

