# Supplementary material for: Mammary gland, kidney and rumen urea and uric acid transporters of dairy cows differing in milk urea concentration
Source: Sci Rep. 2023 Oct 11;13:17231. doi: 10.1038/s41598-023-44416-9 (PMC10567808; doi:10.1038/s41598-023-44416-9)
Supplement: Supplementary file 1 — Supplementary Table S1. [file 41598_2023_44416_MOESM1_ESM.docx]

**Supplemental Material:** Prahl et al. Mammary gland, kidney and rumen urea and uric acid transporters of dairy cows differing in milk urea concentration.

**Supplemental Table 1.** Gene name, primer sequences, amplicon sizes and efficiencies used for real-time RT-PCR.

| Gene^1^ | Primer sequences | Amplicon length [bp] | PCR eﬃciency [%] | References |
| --- | --- | --- | --- | --- |
|  |  |  |  |  |
| *AQP3* | F: GTCCACCACCTCCCACTAGA | 196 | 1.82 | Kuzmany et al.^27^ |
|  | R. TATCTGGAAAACCACGCACA |  |  |  |
| *AQP7* | F: ATTGTGACTGGCATCCTTG | 139 | 1.79 | Sauerwein et al.^28^ |
|  | R: TGGTTCTGAAGACTTGTGAG |  |  |  |
| *AQP10* | F: TCCTGGCCGACATGCTATC | 101 | 1.85 | Røjen et al.^17^ |
|  | R: GCCCCAGCCAGCTACGTA |  |  |  |
| *SLC14A1* | F: AGGGCTACAACGCTACCCTGGTGG | 371 | 1.88 | Coyle et al.^26^ |
|  | R: GAAGATGCCCCCTGTCCACGG |  |  |  |
| *SLC14A2* | F: CCTGGACTGGAGGCATTTTC | 158 | 1.64 |  |
|  | R: CTGTTGAAGCCACATAGTCCG |  |  |  |
| *SLC22A12* | F: GCAGGAAGAGCTGAGTGTGA | 150 | n.d. |  |
|  | R: GGTCCAGGATCAGGCCATAG |  |  |  |
| *ABCG2* | F: AAACTTCTGCCCAGGACTCA | 100 | 1.88 |  |
|  | R: TGAGATATCGATGCCCTGGT |  |  |  |
| *XDH* | F: AAGTCACGGCTCTCAGTGT | 192 | 1.83 | Bühler et al.^29^ |
|  | R: CCACAGCATCCACCATTCTT |  |  |  |
|  | R: GCCCCAGCCAGCTACGTA |  |  |  |
| *EIF3K* | F: CCAGGCCCACCAAGAAGAA | 125 | 1.80 | Kadegowda et al.^30^ |
|  | R: TTATACCTTCCAGGAGGTCCATGT |  |  |  |
|  | R: GCCCCAGCCAGCTACGTA |  |  |  |
| *PPIA* | F: GGATTTATGTGCCAGGGTGGTGA  R: CAAGATGCCAGGACCTGTATG | 120 | 1.85 | Bonnet et al.^31^ |
| *EMD* | F: GCCCTCAGCTTCACTCTCAGA  R: GAGGCGTTCCCGATCCTT | 100 | 1.89 | Saremi et al.^32^ |

^1^ *AQP3*, aquaporin 3; *AQP7*, aquaporin 7; *AQP10*, aquaporin 10; *SLC14A1*, solute carrier family 14 member 1; *SLC14A2*, solute carrier family 14 member 2; *SLC22A12*, solute carrier family 22 member 12; *ABCG2*, ATP binding cassette subfamily G member 2; XDH, xanthine dehydrogenase; *EIF3K*; eukaryotic translation initiation factor-3 subunit K; *PPIA*, peptidylprolyl isomerase A; *EMD*, emerin.
